# The first genome assembly of fungal pathogen *Pyrenophora tritici-repentis* race 1 isolate using Oxford Nanopore MinION sequencing

**DOI:** 10.1186/s13104-021-05751-0

**Published:** 2021-08-28

**Authors:** Paula Moolhuijzen, Pao Theen See, Caroline S. Moffat

**Affiliations:** Centre for Crop Disease and Management, School of Molecular Life Sciences, Curtin University, Bentley, WA 6102 Australia

**Keywords:** *Pyrenophora tritici-repentis*, Genome assembly, Oxford Nanopore, Comparative analysis, ToxA, Tan spot, Yellow spot, MinION

## Abstract

**Objectives:**

The assembly of fungal genomes using short-reads is challenged by long repetitive and low GC regions. However, long-read sequencing technologies, such as PacBio and Oxford Nanopore, are able to overcome many problematic regions, thereby providing an opportunity to improve fragmented genome assemblies derived from short reads only. Here, a necrotrophic fungal pathogen *Pyrenophora tritici-repentis* (Ptr) isolate 134 (Ptr134), which causes tan spot disease on wheat, was sequenced on a MinION using Oxford Nanopore Technologies (ONT), to improve on a previous Illumina short-read genome assembly and provide a more complete genome resource for pan-genomic analyses of Ptr.

**Results:**

The genome of Ptr134 sequenced on a MinION using ONT was assembled into 28 contiguous sequences with a total length of 40.79 Mb and GC content of 50.81%. The long-read assembly provided 6.79 Mb of new sequence and 2846 extra annotated protein coding genes as compared to the previous short-read assembly. This improved genome sequence represents near complete chromosomes, an important resource for large scale and pan genomic comparative analyses.

## Introduction

The necrotrophic fungal pathogen *Pyrenophora tritici-repentis* (Ptr) is the causal agent of tan (or yellow) spot a major disease of wheat (*Triticum aestivum*) [1]. A number of genomic sequencing projects have been undertaken for Ptr [2–6], the majority derived solely from Illumina sequence. Many of these short-read assemblies are incomplete as many genomic regions in Ptr contain long repetitive regions and identical gene copies that are not resolved by short reads [5]. We therefore undertook the currently more affordable Oxford Nanopore Technologies (ONT) long-read sequencing of an Australian Ptr isolate 134 (Ptr134) that was previously sequenced by short read (150 bp paired end) Illumina technology [3].

## Main text

### Methods

#### Isolate collection and sequencing

The pathogenic isolate Ptr134 was isolated from tan spot infected leaves collected from Queensland, Australia in 2001. Ptr134 was cultured in vitro from a single spore [7]. Ptr134 genomic DNA was extracted from 3-day old mycelia grown in vitro in Fries 3 liquid medium, using DNeasy Plant Mini Kit (Qiagen, Hilden, Germany). DNA was further treated with phenol/chloroform extraction, followed by precipitation with sodium acetate and ethanol, and finally resuspension in TE buffer [3]. The Ptr134 genomic DNA was sequenced using a MinION (MIN-101B) Oxford Nanopore StarterPack, R9 (FLO-MINSP6) flow cell, flow cell priming kit (XP-FLP001) and Rapid Sequencing Kit SQK-RAD004, following manufacturers (Oxford Nanopore Technologies, Oxford, UK) protocol. ONT sequencing after 24 h yielded 4,37,865 passed long reads with a total length of 2.6 Gb (65 × genome coverage), base called in real time using MinKNOW version 127.0.0.1 software on a MacBook Pro (version 10.13.6, 2.6 GHz Intel Core i7 processor and 16 GB 2400 MHz DDR4 memory) to a 1 TB Seagate Backup Plus Slim portable storage device (model SRC0VN2), at the Centre for Crop Disease and Management, Perth, Western Australia. ONT sequence data was based called in real time using the MinKNOW Fast basecalling model from Fast5 into FastQ file format. Raw reads were classed as passed by MinKNOW based on the average read quality score > 7. The Ptr134 genome was also previously sequenced via Illumina HiSeq stranded (150 bp paired end reads) by Novogene Co., Ltd (Hong Kong) to yield 3.2 Gb at 80× coverage [3]. The median and maximum read lengths obtained from the MinION were 4253 bp and 91,723 bp, respectively.

#### Genome assembly of Ptr134

The passed FastQ data was error-corrected and assembled using linux-amd64 Canu 1.8 software [8] guided by a genome size of 40 Mb and option for raw nanopore data. Illumina PE reads were quality trimmed for random hexamer primers on the 5′ read end using Trimmomatic v0.22 [9]. The high quality trimmed Illumina reads were aligned to the Canu genome assembly using BWA 0.7.14-r1138 [10] and filtered for concordant PE read alignments using samtools 0.1.19-96b5f2294a [11]. The genome assembly was then corrected with the high quality Illumina alignments using Pilon 1.23 [12] to generate a final polished Ptr134 sequence assembly with 2407 SNPs, 1,64,237 small insertions (totalling 208,176 bases) and 123 small deletions (totalling 151 bases) corrected. Post Canu and Pilon error corrections, the average weighted Phred score base qualities for Ptr134 ONT sequence and a previously PacBio RSII sequenced M4 isolate [3] were 36 and 37, respectively.

Ptr134 was then aligned to M4 [3] scaffolded chromosomes using NUCmer [13] v3.1 (-maxmatch -coords).

#### Gene prediction and functional annotation

Ptr134 Illumina RNA-seq data [3] was aligned to the Ptr134 nanopore assembled genome using TopHat v2.0.12 [14] (-N 2 -i 10 -I 5000 -p 16 –no-discord- ant –no-mixed –report-secondary-alignments –micro- exon-search –library-type fr-firststrand) for supporting ab initio gene predictions by CodingQuarry v1.2 [15] in pathogen mode (PM). Ab initio gene predictions were also made with GeneMark-ES v4.33 [16].

Pt-1C-BFP [2] and M4 reference proteins [3] were aligned to Ptr134 using Exonerate v2.2.0 [17] (–showvulgar no –showalignment no –minintron 10 –maxintron 3000) in mode protein2genome. The ab initio gene predictions and exonerate alignments were then combined using EvidenceModeller v1.1.1 [18] with a minimum intron length of 10 bp and weightings of CodingQuarry:1, GeneMark.hmm:1, protein exonerate:2.

Gene annotations were assigned by BLASTX [19, 20] v2.3.0 + searches across NCBI RefSeq and NR (taxon = Ascomycota) (February 2020) databases and RPSTBLASTN v2.7.1 + of COG, Pfam, Smart and CDD domain databases (February 2020). Final gene annotations were summarised by AutoFACT v3.4 [21]. BUSCO [22] v5.1.2 analysis was conducted on predicted protein sequences using the lineage for pleosporales_odb10.

The ONT Ptr134 annotated genome has been deposited with DDBJ/ENA/GenBank under the updated accession MVBF02000000.

### Results and discussion

#### Genome assembly and annotation of Ptr134

The Ptr134 genome assembled into 28 contiguous sequences with of total length 40.79 Mb and GC content of 50.81% (Table 1). Ptr134 ONT (Version 2) contig length statistics showed marked improvements in comparison to the short-read assembly (Version 1) [3]. In comparison to the previous short read assembly, the long-read assembly provided 6.79 Mb of new sequence. A total of 13,918 protein coding genes were also predicted for the Ptr134 ONT assembly, 2,846 more than the previous short read assembly (Table 1). Although there was no improvement in the BUSCO scores for predicted protein coding genes the new predictions are possible pathogen specific genes found in the more complex regions which are harder to assemble with short reads. The ONT Ptr134 annotated genome has been deposited with DDBJ/ENA/GenBank under the updated accession MVBF02000000 (Table 1).

**Table 1:** *Pyrenophora tritici-repentis* race 1 isolate Ptr134 Oxford Nanopore genome information and assembly statistics compared to race 1 isolate M4 and version 1 short read assembly of Ptr134

	Ptr134 Version2	^a^M4	^a^Ptr134 Version1
Isolate information			
Sequencing Platform	Oxford Nanopore	PacBio RSII and BioNano Optical Map	Illumina (150 bp paired end)
Genome accession	MVBF02000000	NQIK00000000	MVBF01000000
Collection site	Queensland, Australia	Western Australia, Australia	Queensland, Australia
Collection year	2001	2009	2001
Date sequenced	2019	2017	2017
Contig assembly statistics			
Total length (Mb)	40.7	40.9	34.0
Number	28	51	3579
N50 (Mb)	2.687	2.930	0.064
Mean (Kb)	1456	802	9.5
Max (Mb)	6.50	5.60	0.29
GC %	50.1	50.7	50.8
Predicted genes			
Protein coding genes	13,918	13,797	11,072
% Complete ^b^BUSCO	94.3	92.1	94.3

^a^Previously published genome assemblies

^b^Benchmarking Universal Single-Copy Orthologs (BUSCO)

**Fig. 1:**
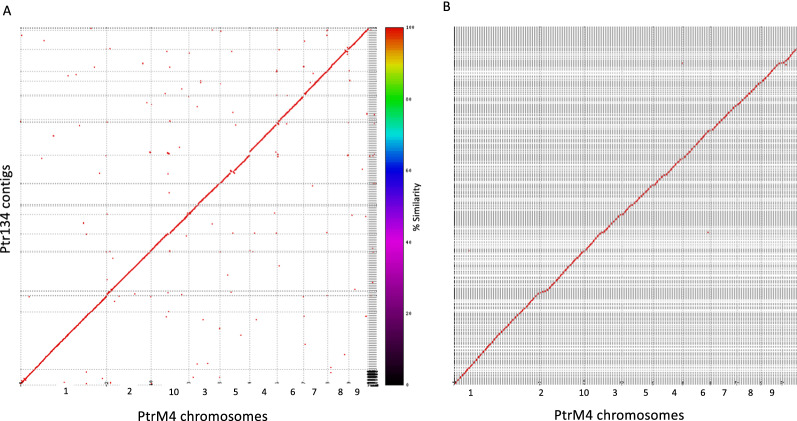
**A** Ptr134 Oxford Nanopore Technology contiguous genome sequences (vertical axis) aligned in a dot matrix plot to M4 assembled chromosomes (horizontal axis). **B** Ptr134 Illumina contiguous genome sequences (vertical axis) aligned in a dot matrix plot against M4 assembled chromosomes (horizontal axis)

The improved Ptr134 genome assembly contains many near complete chromosomes (chromosomes 2, 4, 5, 6, 8, and 9) (Fig. 1). Whole genome alignment of Ptr134 version 2 (Fig. 1A) and Ptr134 version 1 [3] (Fig. 1B) to M4 [3] (PacBio RSII) showed few large-scale rearrangements. However, distinct smaller rearrangements were more clearly observed in the ONT assembly, as compared to the Illumina assembly, in particular a small central sequence inversion in chromosome 5 (Fig. 1A). Furthermore, sequence breaks in Ptr134 relative to M4 chromosomes 1, 3, 7 and 10 reflect sequence variations between the two isolates. In particular, the Ptr134 sequence break relative to M4 chromosome 10 coincides with the chromosome 10 and 11 fusion site revealed previously by optical mapping of M4 [3].

This is the first ONT sequenced, assembled and annotated genome for a Ptr race 1 isolate. The improved ONT genome assembly of Ptr134, over the former Illumina assembly, will enable the better characterization of important genes involved in pathogenicity that are often contained in highly complex genomic regions [5], and contribute to improved pan genomic analyses of this important fungal pathogen.

We demonstrate that ONT is a viable option for sequencing less fragmented and near complete genome assemblies for fungal species. Using these methods researchers can sequence and assemble ‘in house’ isolates of interest to create quality reference genomes.

## Limitations

All methods have been made as consistent as possible for comparative analyses, this analysis has used databases, software and PacBio sequencing versions currently available, which may be updated in the future. The comparison of the two Australian long-read assemblies is only an indication of potential genome stability in Australia.

## Data Availability

The assembled and annotated genome for isolate Ptr134 described in this Data Note can be freely and openly accessed at DDBJ/ENA/GenBank repository under Accession Number- https://www.ncbi.nlm.nih.gov/nuccore/MVBF00000000 (whole genome project) [23].

## Abbreviations

BUSCO: Benchmarking Universal Single-Copy Orthologs
CDD: Conserved Domain Database
COG: Clusters of Orthologous Groups
DDBJ: DNA Data Bank of Japan
ENA: European Nucleotide Archive
Kb: Kilo bases
Mb: Mega bases
NCBI: National Centre for Biotechnology Information
NR: Non-redundant
Pfam: Protein families
ONT: Oxford Nanopore Technologies
SMART: Simple Modular Architecture Research Tool
SNP: Single nucleotide polymorphism
